# Estimating induced abortion incidence and the use of non-recommended abortion methods and sources in two provinces of the Democratic Republic of the Congo (Kinshasa and Kongo Central) in 2021: results from population-based, cross-sectional surveys of reproductive-aged women

**DOI:** 10.1080/26410397.2023.2207279

**Published:** 2023-05-22

**Authors:** Pierre Akilimali, Caroline Moreau, Meagan Byrne, Dynah Kayembe, Elizabeth Larson, Suzanne O. Bell

**Affiliations:** a Professor, Kinshasa School of Public Health, University of Kinshasa, Kinshasa, DRC; b Associate Professor, Department of Population, Family and Reproductive Health, Johns Hopkins Bloomberg School of Public Health, Baltimore, MD, USA; c Soins Primaires et Prévention, CESP Centre for Research in Epidemiology and Population Health, U1018, Inserm, Villejuif F-94800, France; d Senior Program Officer, Department of Population, Family and Reproductive Health, Johns Hopkins Bloomberg School of Public Health, Baltimore, MD, USA.; e Field Coordinator, Kinshasa School of Public Health, University of Kinshasa, Kinshasa, DRC; f PhD Student, Department of Population, Family and Reproductive Health, Johns Hopkins Bloomberg School of Public Health, Baltimore, MD, USA; g Assistant Professor, Department of Population, Family and Reproductive Health, Johns Hopkins Bloomberg School of Public Health, Baltimore, MD, USA.

**Keywords:** Abortion incidence, abortion safety, Democratic Republic of the Congo, survey research, indirect measurement

## Abstract

The changing abortion legal and practice landscape in the DRC in recent years calls for a re-examining of induced abortion experiences. The current study provides population-level estimates of induced abortion incidence and safety by women’s characteristics in two provinces using direct and indirect approaches to assess indirect method performance. We use representative survey data on women aged 15–49 in Kinshasa and Kongo Central collected from December 2021 to April 2022. The survey had questions on respondents’ and their closest friends’ experience with induced abortion, including methods and sources used. We estimated one-year abortion incidence and proportion using non-recommended methods and sources overall and by background characteristics for each province separately for respondents and friends. The fully adjusted one-year friend abortion rate was 105.3 per 1000 women of reproductive age in Kinshasa and 44.3 per 1000 in Kongo Central in 2021; these were substantially higher than corresponding respondent estimates. Women earlier in their reproductive lifespan were more likely to have had a recent abortion. Approximately 17.0% of abortions in Kinshasa and one-third of abortions in Kongo Central involved non-recommended methods and sources according to respondent and friend estimates. The more accurate friend abortion incidence estimates indicate that women in the DRC often rely on abortion to regulate their fertility. Many use non-recommended means and sources to terminate, thus, significant work remains to actualise the commitments made in the Maputo Protocol to provide comprehensive reproductive health services that combine primary and secondary prevention services to reduce unsafe abortion and its consequences.

## Résumé

Les changements de l’environnement juridique et pratique de l’avortement en RDC ces dernières années exigent un réexamen des expériences de l’interruption de grossesse. La présente étude estime au niveau de la population l’incidence des avortements provoqués et la sécurité de la procédure selon les caractéristiques des femmes dans deux provinces, en utilisant des approches directes et indirectes pour évaluer la performance des méthodes indirectes. Nous utilisons des données représentatives d’une enquête auprès de femmes âgées de 15 à 49 ans à Kinshasa et au Kongo-Central collectées en décembre 2021-avril 2022. L’enquête comprenait des questions sur l’expérience des répondantes et de leurs amies proches en matière d’avortement, notamment les méthodes et les sources utilisées. Nous avons estimé l’incidence des avortements sur un an et la proportion des femmes ayant utilisé les méthodes et sources recommandées, de manière globale et en fonction de leurs caractéristiques, pour chaque province séparément pour les répondantes et leurs amies. Le taux d’avortement des amies totalement ajusté sur un an était de 105.3 pour 1000 femmes en âge de procréer à Kinshasa et de 44.3 pour 1000 au Kongo-Central en 2021; ces chiffres étaient nettement plus élevés que les estimations correspondant aux répondantes. Les femmes au début de leur cycle de vie reproductive avaient plus de probabilités d’avoir récemment avorté. Près de 17.0% des avortements à Kinshasa et un tiers des avortements au Kongo-Central avaient eu recours à des méthodes et sources non recommandées d’après les estimations relatives aux répondantes et aux amies. Les estimations plus précises de l’incidence de l’avortement chez les amies indiquent que les femmes en RDC se servent souvent de l’avortement pour réguler leur fécondité. Beaucoup utilisent des méthodes et sources non recommandées pour interrompre leur grossesse; par conséquent il reste beaucoup à faire pour actualiser les engagements souscrits dans le protocole de Maputo en vue d’assurer des services de santé reproductive complets qui combinent les services de prévention primaires et secondaires afin de réduire les avortements à risques et leurs conséquences.

## Resumen

El cambiante panorama legislativo y de prácticas de aborto en la República Democrática del Congo (RDC) en años recientes exige una reexaminación de las experiencias de aborto inducido. El estudio actual ofrece estimaciones de la incidencia y seguridad del aborto inducido a nivel poblacional por características de las mujeres en dos provincias que utilizan enfoques directos e indirectos para evaluar el rendimiento por el método indirecto. Utilizamos datos representativos de una encuesta administrada a mujeres de 15 a 49 años en Kinshasa y Congo Central, recolectados entre diciembre de 2021 y abril de 2022. La encuesta contenía preguntas sobre la experiencia de aborto inducido de las encuestadas y sus amigas más cercanas, incluidos los métodos y fuentes utilizados. Estimamos la incidencia y el porcentaje de abortos durante un año, utilizando métodos y fuentes recomendados en general y por características de antecedentes para cada provincia por separado para las encuestadas y sus amigas. La tasa de aborto completamente ajustada de las amigas en un año fue de 105.3 por cada 1000 mujeres en edad reproductiva en Kinshasa y de 44.3 por cada 1000 en Congo Central en 2021; éstas fueron considerablemente más elevadas que las estimaciones correspondientes para las encuestadas. Las mujeres en las etapas más tempranas de su vida reproductiva eran más propensas a haber tenido un aborto reciente. Para aproximadamente el 17.0% de los abortos en Kinshasa y una tercera parte de los abortos en Congo Central se utilizaron métodos y fuentes no recomendados, según las estimaciones para las encuestadas y sus amigas. Las estimaciones más precisas de incidencia del aborto entre las amigas indican que las mujeres en la RDC a menudo dependen del aborto para regular su fertilidad. Muchas utilizan medios y fuentes no recomendados para interrumpir el embarazo; por ello, aún queda mucho trabajo importante por realizar para actualizar los compromisos asumidos en el Protocolo de Maputo con el fin de proporcionar servicios integrales de salud reproductiva que combinen los servicios de prevención de atención primaria y secundaria para reducir la tasa de abortos inseguros y sus consecuencias.

## Background

Induced abortion is a common reproductive health event globally, with approximately 39 abortions per 1000 women* aged 15–49 annually.^1^ Measuring abortion incidence is important for understanding pregnancy and fertility patterns, but abortion also has public health significance as nearly half of all abortions each year are considered unsafe.^2^ Induced abortion is extremely safe when performed according to medical guidance, however, unsafe abortion is responsible for an estimated 8% of maternal mortality worldwide.^3^ The majority of these deaths occur in the Global South, including many countries in sub-Saharan Africa where a large proportion of abortions occur outside the formal healthcare system due to legal restrictions, stigma, cost, and limited availability or accessibility of safe abortion services.^4^

The changing abortion legal and practice landscape in the Democratic Republic of the Congo (DRC) in recent years calls for an examination of abortion incidence and safety patterns in the country to monitor progress and guide ongoing reforms. It also necessitates the use of recent measurement innovations to better capture the diversity of abortions experienced outside the formal health care sector, ranging from safe self-managed medical abortions to abortions involving non-recommended methods with a higher risk of morbidity and mortality. Up until 2018, abortion was prohibited in the DRC.^5^ The most recent studies of abortion incidence and safety are from this more restrictive period, when investigators from different studies estimated an annual abortion incidence of 55 and 56 abortions per 1000 women aged 15–49 in the capital, Kinshasa, in 2015 and 2016, respectively.^6,7^ Many of these induced abortions led to complications and required post-abortion care for treatment. One community-based study in Kinshasa suggested half of the abortions resulted in complications, while findings from this and another study indicate only 26% to 39% of women who had an abortion sought post-abortion care for treatment of potential complications ^7,6^ Other research found that 15% of obstetric and gynaecology emergency room inpatients were experiencing complications due to unsafe induced abortion, 6% of whom died.^8^ An estimated 88% of abortions are unsafe in the Central African region,^2^ and unsafe abortion is a significant contributor to maternal morbidity and mortality, responsible for an estimated 10% of maternal deaths in sub-Saharan Africa.^3^

These alarming statistics led to unprecedented political action to reduce unsafe abortion-related morbidity and mortality in the DRC, which officially decriminalised induced abortion in 2018 by codifying their ratification of the African Union’s Maputo Protocol into law.^9,10^ The treaty allows for legal abortion access in cases of sexual assault, rape, incest, fetal abnormalities, and when continuing the pregnancy endangers the mental or physical health or life of the pregnant person. The Ministry of Health subsequently approved comprehensive abortion care guidelines in alignment with the Maputo Protocol in 2020, which made abortion legal under the previously specified conditions up to 14 weeks of pregnancy and removed key barriers to accessing safe abortion, including parental consent for minors, spousal consent for married women, and proof of rape claims. The guidelines also allow for task shifting to mid-level providers at any facility with adequate equipment, including nurses and midwives, widening the range of providers and facilities that can offer this care. Such legal reforms are promising but implementation of new laws may be slow and result in delayed improvements in abortion safety, as seen in other settings.^11,12^ A weakened health system, abortion costs, and social stigma surrounding abortion in the DRC may further hamper progress.^13,14^ Many pregnant people may forego facility-based safe abortion options to avoid being seen at a facility and risk others learning of their abortion.^15^ With the diffusion of medical abortion pills outside of the formal healthcare system offering a safe self-management option to privately terminate a pregnancy, non-facility-based pregnancy termination may become more enticing to those with great concerns regarding threats to their social safety.^16^ This has significant implications for measuring and monitoring this phenomenon.

Despite the demographic and public health importance of measuring abortion incidence and safety, it is quite challenging. One of the prior studies of abortion incidence and safety in Kinshasa relied on facility-derived post-abortion complication treatment rates, which investigators adjusted to account for abortions that do not present for post-abortion care.^6^ This method, known as the Abortion Incidence Complication Method (AICM), has been used extensively in the Global South where researchers cannot rely on facility-based logbook data due to pregnant people’s extensive use of informal sources of care and self-managed abortion amid legal restrictions and stigma.^17^ In recent years, researchers at the organisation that developed this method have made adjustments to the method and explored the use of other approaches, given concerns about the validity of the adjustments made to account for abortions taking place entirely outside the formal sector with the growing use of safe, self-managed medication abortion.^18–21^

One promising methodological area of research is social network-based approaches to measure sensitive behaviours, like abortion, which ask respondents to report on friends’ sensitive behaviours rather than their own, to reduce the social desirability bias and associated under-reporting observed with direct questions.^22,23^ Different versions of this method have been used, with varying numbers of friends (one, two, or three) and different relationship criteria (mutual sharing of sensitive information or just closest friend(s)).^24–26,31^ Most studies using this indirect population-based approach yield higher abortion estimates than direct estimates based on self-reports, although the performance of the methodology has varied by context.^18,21,27–32^ Several studies employing these social network-based techniques have also found similar abortion characteristics for respondents and friends or friends and facility-based data, though friend estimates are often slightly less safe than those of respondents, suggesting greater visibility of less safe abortions.^22,27–29,32^ These social network-based approaches rely on three main assumptions: (1) friends are similar to respondents (no selection bias), (2) respondents know about their friends’ sensitive behaviours (no transmission bias), and (3) respondents report their friend’s sensitive behaviours more accurately than their own (no social desirability bias). One of the 2016 studies examining abortion incidence in Kinshasa used a version of this method, asking respondents to report on their closest friends’ abortion experiences. This methodology seemed to perform well based on respondents’ ability to report on their confidants’ characteristics and abortions and the resulting abortion incidence, which was similar to the other contemporaneous estimate produced via the AICM.^7^ However, the study did not examine or make adjustments to account for potential violations of method assumptions, nor did they compare estimates to within sample direct reports as a form of internal validation.^7^

The current study provides updated and expanded population-level information on the incidence of induced abortion and use of non-recommended methods and sources of abortion by women’s socio-demographic characteristics following abortion decriminalisation in Kinshasa and provides a point of comparison in Kongo Central, a more rural area of the country where we might expect abortion patterns and termination methods to differ. In pursuit of this aim we evaluate the performance of a social network-based method where we ask about respondents’ closest female friends aged 15–49 living in the country and adjust friend abortion estimates for potential selection and transmission bias. We refer to this method as the “best friend method”. We present the respondent abortion estimates to assess method performance and triangulate data on abortion patterns.

## Methodology

### Sampling and data collection

We used data from the Performance Monitoring for Action (PMA) study conducted in Kinshasa and Kongo Central between December 2021 and April 2022. PMA conducts nationally or regionally representative surveys on reproductive health in nine countries in Africa and Asia.^33^ PMA DRC conducts surveys in two provinces, Kinshasa and Kongo Central. We used a two-stage cluster sampling design in each province. We selected clusters using probability proportional to size sampling, listed all households within each cluster, and randomly selected 35 households per cluster to identify a representative sample of households. All women aged 15–49 identified in each household survey roster were invited to participate in the female survey in 2019–20 (Phase 1). These women were followed annually in 2020–21 (Phase 2) and 2021–22 (Phase 3). A total of 2611 women in Kinshasa and 1950 women in Kongo Central were selected at baseline (response rates of 95.3% and 98.7%, respectively). The final PMA Phase 3 sample used in this study included 1554 and 1266 women from the panel and 772 and 590 women from replacement households to account for attrition in order to produce cross-sectional estimates based on the *de facto* population of reproductive-aged women. Altogether, the current analysis includes 2326 reproductive-aged women from Kinshasa and 1856 from Kongo Central. More details on PMA selection procedures can be found at www.pmadata.org/data/survey-methodology. Ethical approval for this study was obtained from the Institutional Review Board at the Johns Hopkins University Bloomberg School of Public Health (#14590) in November 2021 and the Comité d’Éthique at the Kinshasa School of Public Health (#ESP/CE/159B/2021) in October 2021.

After providing verbal informed consent, women were invited to respond to a face-to-face questionnaire soliciting information on women’s socio-demographic characteristics and fertility intentions and behaviours, including contraceptive use. In the DRC Phase 3 female survey, we added an abortion module derived from prior PMA abortion work in Nigeria, Côte d’Ivoire, and Rajasthan, India^27–29,34^ to explore respondents’ and their closest friends’ experience with abortion. Interviewers first asked respondents to identify their closest female friend aged 15–49 currently living in the DRC. They subsequently described their friend’s characteristics before being asked about their friend’s abortion experiences and their own abortion experiences. We used both the respondent data and surrogate sample produced by the friend data to examine abortion characteristics and patterns in our analyses.

### Measures

Abortion experiences (for friends and respondents) were measured using two sets of questions; one asking about ever doing something to end a pregnancy and another about ever doing something to bring back a late menstrual period. For menstrual regulation, we asked a follow-up question to clarify whether the action they took was because they were worried they were pregnant. In this study, abortion included all successful attempts to intentionally end a pregnancy and all successful attempts to intentionally bring back a period when worried about being pregnant. Subsequent questions examined when the most recent abortion experience occurred, the method(s) and source(s) used, and whether there were complications.

We used data on reported abortion source(s) and method(s) to create a categorical measure of whether the abortion involved a recommended method and/or source. To do so we adapted the latest World Health Organization (WHO) indicator of abortion safety, which defines safe abortion as one conducted by trained providers using recommended methods (i.e. abortion surgery or misoprostol with or without mifepristone), less safe abortions as those involving *either* appropriately trained providers *or* a recommended method, and least safe abortions involving non-recommended methods and conducted by untrained providers.^2^ Based on our data – which contain no information on provider training – we considered abortions from public or private clinical sources (national hospital, regional hospital, government health centre, family planning clinic, maternity, community health worker, private hospital, NGO, private health centre, private practice, private doctor, mobile nurse, community health agents) as involving a recommended source. All other sources were considered non-recommended with the exception of sources of medical abortion pills. To account for the WHO’s latest abortion guidelines that consider self-managed medical abortion to be safe under certain conditions, we categorised all abortions using medical abortion pills (i.e. misoprostol with or without mifepristone) from any source as involving a recommended source.^35^ We have no information on the details of the surgery or medical abortion pill regimen, however, we classified surgical abortion and medical abortion pills as recommended methods and all other methods (other or unknown pills, injections, traditional methods) as non-recommended. Our categories thus assess whether the abortion involved a “recommended method and source”, a “non-recommended method or source”, or a “non-recommended method and source”.

We considered the following socio-demographic and reproductive health indicators for respondents and friends: age, education, marital status, province of residence (assuming the friend is in the respondent’s province), parity, current use of contraception, and current long-acting reversible contraceptive (LARC) use, which includes intrauterine devices (IUDs) and implants. We examined the prevalence of LARC use specifically as respondents and friends might be able to more accurately report on the use of these long-term methods compared to a behavioural, coital-dependent, or fully-user-controlled method that a woman may stop and start more frequently. Other researchers have suggested comparing LARC prevalence among respondents and friends as a validation of social network-based method assumption of surrogate sample similarity and the sharing of less sensitive reproductive health behaviours.^20^ We also examined wealth tertiles for respondents, which we derived from a continuous wealth measure using principal components analysis from information on household assets, water, sanitation, and building materials following a similar method to that employed by the Demographic and Health Surveys; we were unable to obtain these household details for friends and thus we cannot examine abortion patterns by wealth for the surrogate sample.

### Analysis

To examine and adjust for violations of assumptions (selection and transmission biases) in the friend data, we followed a five-step process:

1. We examined the proportion of respondents who reported having no close friends and tested for socio-demographic and reproductive differences between respondents who had friends and those who did not use design-based *F* tests.
2. Given the observed differences in respondents who did and did not report having a friend, we incorporated respondents who reported having no close friends in the DRC into the surrogate friend sample since women without friends are essentially “missing” from the unadjusted surrogate sample. This included incorporating data on these respondents’ abortion methods and sources.
3. In order to generate a more accurate likelihood that a respondent with no friends had an abortion in the past year given under-reporting of abortion via direct question,^23^ we used the adjusted surrogate sample (described in Step 2) to regress socio-demographic characteristics on abortion incidence while excluding the self-reported abortion incidence data for the respondents with no friends in this sample. We then used the predicted probability of abortion incidence from this model to generate the likelihood of a respondent with no friends in the adjusted surrogate sample having had an abortion in the prior year.
4. To align the adjusted surrogate sample’s socio-demographic distribution with the respondent sample’s characteristics, which were designed to be representative of reproductive-aged women in each province, we applied post-stratification weights. To calculate these weights, we regressed the respondent and surrogate sample socio-demographic characteristics on whether the observation was a respondent from the original sample or an observation in the adjusted surrogate sample and took the inverse of the predicted probability of being in the adjusted surrogate sample. We multiplied these weights by the survey design weights and applied them when analysing the adjusted surrogate sample. The post-stratification weights ultimately had little impact on abortion estimates as the magnitude of differences between respondent and adjusted surrogate sample socio-demographic characteristics were small.
5. Given respondent knowledge of their friend’s abortions is likely incomplete, we needed to account for this transmission bias in the friend abortion incidence estimates. To do so we asked respondents who reported their own abortion and had a close female friend, “Do you think your friend, [fake name of friend], knows about this event?”, with response options “Yes”, “Maybe”, “No”, and “Do not know”. Those who replied “Yes” or “Maybe” were subsequently asked *how* the friend knows. We then calculated the transmission bias adjustment factor as the inverse proportion of respondents who reported directly sharing their own abortion experience with their closest friend.^18,30,21^ This adjustment assumes sharing patterns were similar in both directions (respondent to friend and friend to respondent). We only included friend abortions that the respondent indicated the friend had directly told her about to improve the likelihood of this assumption. Given different sharing patterns by whether the abortion was reported as ending a pregnancy or bringing back a late period, we adjusted for the relative distribution of these phenomena in each province and the corresponding proportion of respondents who shared the experience with their closest friend in calculating the transmission bias adjustment factor for each province.

Using these adjustments, we compared the respondent’s one-year abortion incidence to the friend’s adjusted incidence, overall and by socio-demographic characteristics. We included abortions reported in 2021 and early 2022 to account for potential displacement across years. We computed one-year abortion incidence rates for respondents and friends by dividing all abortions reported in 2021 and early 2022 by the average number of person-years between January 2021 and the date of interview, which was in December 2021 or early 2022. Given the timing of fieldwork, this average was 1.03 years in Kinshasa and 1.05 years in Kongo Central. We then multiplied the rate by 1000. We compared the fully adjusted friend estimates to respondent rates by assessing whether confidence intervals overlapped, given no suitable statistical test was found due to the *post hoc* nature of the transmission bias adjustment described above.

Next, we examined the distribution of abortion methods and sources for respondents and friends. We also explored socio-demographic characteristics associated with the use of non-recommended abortion methods and sources, which may pose the greatest risk of negative sequelae, for respondents and friends. We used design-based *F* tests to assess whether differences were statistically significant.

We conducted analyses using Stata version 17.0.^36^ We constructed survey design weights separately for each province to account for the complex sampling strategy, reflecting the inverse probability of selection and non-response and calculated robust standard errors to adjust for clustering. We combined the design weights with the post-stratification weights for the surrogate sample.

## Results

Our sample was comprised of 2326 women in Kinshasa and 1856 in Kongo Central (Tables 1 and 2). The mean age of women was 28.2 in Kinshasa and 29.4 in Kongo Central. Women in Kongo Central were more likely to be married than women in Kinshasa (59.5% versus 41.5%) and more likely to have children (75.8% versus 56.9%). Conversely, women in Kinshasa had a higher level of education than women in Kongo Central (94.5% had secondary education or higher versus 65.0%). A similar proportion of women were using contraception at the time of the survey (41.5% in Kinshasa and 39.1% in Kongo Central), around one-quarter of which was LARCs (8.2% in Kinshasa, 9.9% in Kongo Central). Altogether approximately 64% of women in Kinshasa and Kongo Central reported having a close female friend living in the DRC (1427 in Kinshasa and 1184 in Kongo Central). Women with friends were more likely to be married and have children in Kinshasa, while they had less education and were less likely to be using LARCs in Kongo Central.

**Table 1.** Characteristics of female respondents aged 15–49 overall and by whether they reported having a close female friend in Kinshasa, DRC*

	All respondents		0 Friends		≥ 1 Friend	
	%	*N*	%	*N*	%	*N*
Age						
15–19	22.3	516	*21.5*	*183*	*22*.*7*	*333*
20–29	37.9	881	*35*.*6*	*324*	*39*.*1*	*557*
30–39	23.5	558	*23*.*2*	*218*	*23*.*6*	*340*
40–49	16.4	371	*19*.*7*	*174*	*14*.*6*	*197*
Education						
Never/Primary	5.5	145	*6*.*2*	61	*5*.*2*	84
Secondary	72.6	1696	*75*.*2*	668	*71*.*2*	1028
Tertiary	21.9	485	*18*.*6*	170	*23*.*6*	315
Currently married						
No	58.5	1353	**53**.**4**	482	**61**.**3**	871
Yes	41.5	973	**46**.**6**	417	**38**.**7**	556
Wealth tertile						
Poorest	29.9	780	31.8	301	28.9	479
Middle wealth	32.9	785	35.1	323	31.7	462
Wealthiest	37.2	761	33.0	275	39.4	486
Has any children						
No	43.1	980	**38**.**7**	340	**45**.**4**	640
Yes	56.9	1345	**61**.**3**	559	**54**.**6**	786
Currently using contraception						
No	58.5	1383	*61*.*4*	564	*56*.*9*	819
Yes	41.5	943	*38*.*6*	335	*43*.*1*	608
Currently using LARC						
No	91.8	2149	91.9	829	91.7	1320
Yes	8.2	177	8.1	70	8.3	107
Annual abortion incidence (per 1000)	18.7	46	12.3	8	21.6	38
Total	100.0	2326	100.0	899	100.0	1427

*Estimates weighted, *N*s unweighted; bold indicates *p*-value for design-based *F* test (comparing respondents with 0 friends to those with 1 + friends) less than 0.05

**Table 2.** Characteristics of female respondents aged 15–49 overall and by whether they reported having a close female friend in Kongo Central, DRC*

	All respondents		0 Friends		≥ 1 Friend	
	%	*N*	%	*N*	%	*N*
Age						
15–19	21.1	417	19.7	136	21.9	281
20–29	32.0	598	29.3	192	33.5	406
30–39	27.6	501	28.1	190	27.4	311
40–49	19.2	340	22.9	154	17.2	186
Education						
Never	6.8	116	**9.9**	63	**5**.**0**	53
Primary	28.2	458	**28**.**2**	175	**28**.**3**	283
Secondary/Higher	65.0	1282	**61**.**9**	434	**66**.**7**	848
Currently married						
No	40.5	787	38.0	255	41.9	532
Yes	59.5	1069	62.0	417	58.1	652
Wealth tertile						
Poorest	29.2	403	34.3	178	26.3	225
Middle wealth	32.8	527	31.2	193	33.7	334
Wealthiest	38.0	926	34.5	301	40.0	625
Has any children						
No	24.2	494	22.6	151	25.0	343
Yes	75.8	1361	77.4	521	75.0	840
Currently using contraception						
No	60.9	1095	63.7	418	59.2	677
Yes	39.1	761	36.3	254	40.8	507
Currently using LARC						
No	90.1	1682	**93**.**0**	623	**88**.**5**	1059
Yes	9.9	174	**7**.**0**	49	**11**.**5**	125
Annual abortion incidence (per 1000)	16.9	26	*7.3*	4	*19.9*	22
Total	100.0	1856	100.0	672	100.0	1184

*Estimates weighted, *N*s unweighted; bold indicates *p*-value for design-based *F* test (comparing respondents with 0 friends to those with 1 + friends) less than 0.05.

Friends (based on both the unadjusted and adjusted surrogate sample) were very similar to respondents in both provinces. In Kinshasa, the adjusted friend sample was slightly older than the respondent sample (Table 3). In Kongo Central, friends were slightly less educated and 1.7 percentage points less likely to have children even in the adjusted sample (Table 4). Marital status, current contraceptive use, and LARC use were similar among respondents and friends in both provinces.

**Table 3.** Characteristics of female respondents aged 15–49 and their closest female friends age 15–49 in Kinshasa, DRC*

	Respondent		Unadjusted friend**		Adjusted friend**	
	%	*N*	%	*N*	%	*N*
Age						
15–19	22.3	516	20.9	297	**21.8**	**483**
20–29	37.9	881	35.8	507	**36**.**7**	**842**
30–39	23.5	558	25.4	363	**24**.**0**	**587**
40–49	16.4	371	17.9	236	**17**.**5**	**414**
Education						
Never/Primary	5.5	145	4.3	70	5.4	135
Secondary	72.6	1696	70.3	988	72.1	1666
Tertiary	21.9	485	25.3	355	22.5	525
Currently married						
No	58.5	1353	58.6	827	57.7	1310
Yes	41.5	973	41.4	599	42.3	1016
Wealth tertile						
Poorest	29.9	780	–	–	–	–
Middle wealth	32.9	785	–	–	–	–
Wealthiest	37.2	761	–	–	–	–
Has any children						
No	43.1	980	44.9	635	42.9	976
Yes	56.9	1345	55.1	791	57.1	1350
Currently using contraception						
No	58.5	1383	54.9	788	57.1	1352
Yes	41.5	943	45.1	639	42.9	974
Currently using LARC						
No	91.8	7	**89**.**2**	**1279**	90.1	2108
Yes	8.2	177	**10**.**8**	**148**	9.9	218
Total	100.0	2326	100.0	1427	100.0	2326

*Estimates weighted, Ns unweighted; bold indicates *p*-value for design-based *F* test (comparing friends to respondents) less than 0.05.

**Unadjusted friend estimates calculated directly from respondent reported friend data. Adjusted friend estimates include respondent characteristics in place of “missing” confidantes and apply the post-stratification weights.

**Table 4.** Characteristics of female respondents aged 15–49 and their closest female friends age 15–49 in Kongo Central, DRC*

	Respondent		Unadjusted friend**		Adjusted friend**	
	%	*N*	%	*N*	%	*N*
Age						
15–19	21.1	417	20.3	247	20.7	388
20–29	32.0	598	32.9	387	31.8	595
30–39	27.6	501	28.4	305	28.0	504
40–49	19.2	340	18.4	201	19.5	369
Education						
Never	6.8	116	**11.4**	**114**	**9**.**0**	**177**
Primary	28.2	458	**21**.**7**	**210**	**26**.**2**	**388**
Secondary/Higher	65.0	1282	**66**.**9**	**852**	**64**.**8**	**1291**
Currently married						
No	40.5	787	45.4	539	42.7	795
Yes	59.5	1069	54.6	644	57.3	1061
Wealth tertile						
Poorest	29.2	403	–	–	–	–
Middle wealth	32.8	527	–	–	–	–
Wealthiest	38.0	926	–	–	–	–
Has any children						
No	24.2	494	**28**.**5**	**364**	**25**.**9**	**520**
Yes	75.8	1361	**71**.**5**	**812**	**74**.**1**	**1335**
Currently using contraception						
No	60.9	1095	62.0	684	62.2	1102
Yes	39.1	761	38.0	500	37.8	754
Currently using LARC						
No	90.1	16	*87*.*3*	*1021*	89.2	1644
Yes	9.9	174	*12*.*7*	*163*	10.8	212
Total	100.0	1856	100.0	1184	100.0	1856

*Estimates weighted, Ns unweighted; bold indicates *p*-value for design-based *F* test (comparing friends to respondents) less than 0.05.

**Unadjusted friend estimates calculated directly from respondent reported friend data. Adjusted friend estimates include respondent characteristics in place of “missing” confidantes and apply post-stratification weights.

To adjust for transmission bias, we examined the percentage of respondents who shared their abortion with their closest female friend among those who reported both having an abortion and a close friend. Among this group of women in Kinshasa, 62.2% reported sharing their experience ending a pregnancy with their closest female friend while 51.2% reporting sharing their period regulation with their friend; the corresponding numbers in Kongo Central were 61.7% and 39.7% (estimates not shown). Given 86.3% and 83.1% of abortions were reported as ending a pregnancy (versus bringing back a late period) in these provinces, we used this distribution to calculate the transmission bias adjustments factor, which was the inverse probability of sharing one’s abortion with one’s closest female friend; the adjustment factor was 1.65 in Kinshasa and 1.77 in Kongo Central.

The induced abortion incidence rates were much higher for the friends compared to respondents; however, patterns were similar in both samples (Tables 5 and 6). In Kinshasa, the respondent one-year incidence of induced abortion was 18.7 (95% CI 12.3–25.0) abortions per 1000 women of reproductive age in 2021 while the fully adjusted friend rate was 105.3 (95% CI 78.6–131.9). In Kongo Central, the respondent one-year incidence of induced abortion was 16.9 (95% CI 7.4–26.3) abortions per 1000 women of reproductive age in 2021 whereas the adjusted friend rate was 44.3 (95% CI 23.0–65.7). Across provinces, respondent and friend abortion rates were highest among 20- to 29-year-olds (though they were similarly high for adolescents among friends in Kinshasa), unmarried women, and women without children. In Kinshasa, the abortion rate was highest for respondents with greater education, similar to patterns among respondents and friends in Kongo Central; however, the Kinshasa friend abortion rate was lowest among those with the most education.

**Table 5.** Induced abortion incidence (per 1000) among female respondents aged 15–49 and their closest female friends aged 15–49 in Kinshasa, DRC, in 2021 by background characteristics*

	Respondent (*n* = 2326)			Adjusted friend** (*n* = 2326)		
	Rate	95% CI		Rate	95% CI	
Age						
15–19	21.1	10.9	40.8	**138.9**	**78**.**8**	**199**.**1**
20–29	27.2	18.3	40.5	**138**.**9**	**104**.**1**	**173**.**6**
30–39	12.5	5.8	27.1	**79**.**9**	**50**.**7**	**109**.**1**
40–49	6.9	1.5	32.4	27.7	8.8	46.6
Education						
Never/Primary	12.0	0.0	27.6	**112**.**2**	**29**.**4**	**195**.**0**
Secondary	17.4	10.6	24.2	**110**.**1**	**79**.**7**	**140**.**6**
Tertiary	24.6	9.5	39.6	**88**.**4**	**46**.**5**	**130**.**2**
Currently married						
No	24.1	13.9	34.3	**142**.**3**	**107**.**1**	**177**.**4**
Yes	11.0	4.1	17.9	**54**.**8**	**35**.**5**	**74**.**0**
Wealth tertile						
Poorest	29.4	15.1	43.6	–	–	–
Middle wealth	21.2	11.2	31.2	–	–	–
Wealthiest	8.1	1.7	14.5	–	–	–
Has any children						
No	24.7	12.3	37.1	**118**.**3**	**78**.**0**	**158**.**5**
Yes	14.1	6.8	21.4	**95**.**4**	**71**.**1**	**119**.**8**
Overall	18.7	12.3	25.0	**105**.**3**	**78**.**6**	**131**.**9**

*Estimates weighted, Ns unweighted; bold indicates 95% confidence intervals do not overlap (reference respondents).

**Estimate include respondent characteristics in place of “missing” confidantes; post-stratification weights and transmission bias adjustment applied

**Table 6.** Induced abortion incidence (per 1000) among female respondents aged 15–49 and their closest female friends aged 15–49 in Kongo Central, DRC in 2021, by background characteristics*

	Respondent (*n* = 1856)			Adjusted friend** (*n* = 1856)		
	Rate	95% CI		Rate	95% CI	
Age						
15–19	9.1	2.0	42.8	52.4	14.7	90.2
20–29	23.6	8.3	67.6	61.4	21.8	100.9
30–39	22.8	9.7	53.8	43.9	11.7	76.2
40–49	6.3	1.1	36.7	6.2	4.0	8.5
Education						
Never	11.9	0.0	35.3	4.4	0.0	13.5
Primary	12.1	0.0	24.7	42.8	8.0	77.6
Secondary/Higher	19.2	5.7	32.8	49.9	19.6	80.1
Currently married						
No	29.0	7.7	50.4	61.8	38.4	85.3
Yes	8.4	1.8	15.0	30.6	6.1	55.1
Wealth tertile						
Poorest	18.5	0.0	47.9	–	–	–
Middle wealth	10.5	0.0	22.9	–	–	–
Wealthiest	21.0	7.7	34.3	–	–	–
Has any children						
No	21.0	0.2	41.7	52.8	22.2	83.5
Yes	15.6	7.7	23.5	41.3	19.1	63.5
Overall	16.9	7.4	26.3	44.3	23.0	65.7

*Estimates weighted, Ns unweighted; bold indicates 95% confidence intervals do not overlap (reference respondents).

**Estimates include respondent characteristics in place of “missing” confidantes; post-stratification weights and transmission bias adjustment applied.

The relative frequency with which women relied on specific methods and sources was similar between provinces, but they involved more non-recommended methods in Kongo Central (Tables 7 and 8). While just over one-quarter of respondents used more than one method to end their pregnancy in Kinshasa (26.5%), 44.0% of respondents in Kongo Central used more than one method; estimates were similar for friends in Kinshasa (26.1%) and Kongo Central (38.9%). Surgery was the most common method used among respondents in Kinshasa (42.2%) followed by medical abortion pills (25.5%) while the order was reversed in Kongo Central; 28.3% surgery and 30.7% medical abortion pills. Injection and other non-medical abortion pills were also very common in Kinshasa (19.4% and 13.4%, respectively) and Kongo Central (17.4% and 15.6%, respectively). Private facilities were the most common source of care in Kinshasa at 51.4% followed by pharmacies at 31.6%. In Kongo Central, more relied on pharmacies (39.5%) followed by public (28.7%) and private (27.5%) facilities. The distribution of abortion methods and sources were generally similar among friends in both provinces, though the use of other non-medical abortion pills and public facilities was significantly higher in Kongo Central.

**Table 7.** Details of most recent reported abortion among female respondents aged 15–49 and their closest female friends aged 15–49 in Kinshasa, DRC*

	Respondent (*n* = 307)		Adjusted friend** (*n* = 358)	
	%	*N*	%	*N*
Used multiple methods/sources	26.5	88	26.0	104
All methods used***				
Surgery	42.2	118	51.0	175
Medical abortion pills	25.5	76	23.2	83
Other pills (identified)	13.4	47	15.6	77
Unknown pill type	7.7	27	7.3	23
Injection	19.4	65	13.7	58
Traditional/other methods	9.8	29	12.9	46
Do not know/No response	1.4	6	4.2	14
All sources used***				
Public facility	17.6	50	14.2	53
Private facility	51.4	158	61.7	224
Pharmacy	31.6	98	30.3	113
Other non-clinical	10.6	34	10.5	38
Do not know/No response	**0**.**2**	**1**	**1**.**9**	**8**

*Estimates weighted, Ns unweighted; bold indicates *p*-value for design-based *F* test (reference respondents) less than 0.05.

**Estimates include respondent characteristics in place of “missing” confidantes; post-stratification weights applied.

***Respondents could select multiple methods/sources thus percentages do not sum to 100%.

**Table 8.** Details of most recent reported abortion among female respondents aged 15–49 and their closest female friends aged 15–49 in Kongo Central, DRC*

	Respondent (*n* = 148)		Adjusted friends** (*n* = 156)	
	%	*N*	%	*N*
Used multiple methods/sources	44.0	66	38.9	60
All methods used***				
Surgery	28.3	53	21.6	51
Medical abortion pills	30.7	28	40.0	48
Other pills (identified)	**15.6**	**25**	**29**.**8**	**43**
Unknown pill type	9.6	13	14.8	14
Injection	13.0	25	14.0	36
Traditional/other methods	17.4	26	26.0	34
Do not know/No response	2.2	7	2.3	7
All sources used***				
Public facility	**28**.**7**	**42**	**38**.**8**	**57**
Private facility	27.5	48	34.9	54
Pharmacy	39.5	52	48.8	66
Other non-clinical	15.2	21	17.4	26
Do not know/No response	*0*.*3*	*1*	*1*.*7*	*4*

*Estimates weighted, Ns unweighted; bold indicates *p*-value for design-based *F* test (reference respondents) less than 0.05.

**Estimates include respondent characteristics in place of “missing” confidantes; post-stratification weights applied.

***Respondents could select multiple methods/sources thus percentages do not sum to 100%.

Based on the source and method data, we observed different levels of abortions involving recommended methods and sources in Kinshasa and Kongo Central (Table 9). Abortions that used recommended methods and sources constituted 64.9% and 57.0% of abortions in Kinshasa and Kongo Central, respectively; friend data suggested similar levels (62.2% and 52.2%), neither of which were significantly different from respondent estimates. Approximately 3 in 10 (29.9%) abortions involved non-recommended methods and sources in Kongo Central versus only 16.8% in Kinshasa (Table 9). The corresponding estimates for friends were 34.3% and 17.0%, respectively.

**Table 9.** Use of recommended versus non-recommended methods and/or sources in most recent abortion among female respondents aged 15–49 and their closest female friends aged 15–49 in Kinshasa and Kongo Central, DRC*

	Respondent		Adjusted friend**	
Kinshasa	%	*N*	%	*N*
Recommended method and source***	64.9	187	62.2	212
Non-recommended method or source	18.3	61	20.8	76
Non-recommended method and source	16.8	59	17.0	70
Total	100.0	307	100.0	358
**Kongo Central**				
Recommended method and source***	57.0	78	52.2	78
Non-recommended method or source	13.2	27	13.5	29
Non-recommended method and source	29.9	43	34.3	49
Total	100.0	148	100.0	156

* Estimates weighted, *N*s unweighted; bold indicates statistically significantly difference at the *p* < 0.05 level (reference respondent) based on design-based *F* test.

**Adjusted friend data includes respondent abortion details in place of “missing” confidantes; post-stratification weights applied.

***Surgery from facility and medical abortion pills (misoprostol with or without mifepristone) from any source = recommended method and source.

We combined the province-specific abortion data for sample size considerations to examine patterns in the proportion of abortions that involved non-recommended methods and sources. The extent of abortions using non-recommended methods and sources differed significantly by women’s education in the friend data, with a greater reliance on these methods and sources among those with less education; estimates ranged from 10.0% among those with tertiary education to 64.7% among those with no formal education (Table 10). Respondent and friend data both indicated significantly higher use of non-recommended methods and sources in Kongo Central than in Kinshasa.

**Table 10.** Percent of abortions involving non-recommended methods and sources among female respondents aged 15–49 and their closest female friends aged 15–49 in Kinshasa and Kongo Central, DRC, by background characteristics*

	Respondent (*n* = 455)			Adjusted friend** (*n* = 514)		
	%	95% CI		%	95% CI	
Age						
15–19	24.2	9.1	39.4	31.4	19.9	42.9
20–29	21.7	15.3	28.1	18.8	11.7	25.9
30–39	17.3	10.2	24.4	19.8	9.6	29.9
40–49	24.9	9.7	40.1	28.3	15.7	40.9
Education						
Never	50.8	0.0	112.6	**64.7**	**35**.**8**	**93**.**7**
Primary	27.2	11.7	42.7	**43**.**7**	**19**.**9**	**67**.**5**
Secondary	21.3	15.6	26.9	**22**.**0**	**16**.**4**	**27**.**7**
Tertiary	14.6	4.8	24.4	**10**.**0**	**2**.**4**	**17**.**5**
Currently married						
No	19.8	13.7	25.8	22.2	14.2	30.1
Yes	22.4	13.8	31.0	21.6	14.2	28.9
Wealth tertile						
Poorest	*23*.*9*	*14*.*7*	*33*.*1*	–	–	–
Middle wealth	*13*.*6*	*6*.*9*	*20*.*4*	–	–	–
Wealthiest	*25*.*7*	*16*.*5*	*34*.*9*	–	–	–
Province***				** **	** **	** **
Kinshasa	**17**.**0**	**11**.**5**	**22**.**4**	**17**.**1**	**12**.**0**	**22**.**2**
Kongo Central	**29**.**7**	**18**.**2**	**41**.**2**	**34**.**2**	**18**.**8**	**49**.**7**
Parity						
0	23.2	13.7	32.8	*20*.*9*	*13*.*9*	*27*.*9*
1+	20.3	14.1	26.4	*22*.*5*	*15*.*4*	*29*.*6*
Total	21.0	15.5	26.4	21.9	15.9	27.9

*Estimates weighted, Ns unweighted; bold indicates statistically significantly difference at the *p* < 0.05 level (comparing proportions across categories) based on design-based *F* test.

**Adjusted friend data includes respondent abortion details in place of “missing” confidantes; post-stratification weights applied.

***Estimates differ from those in Table 9 as we could not weight data in this table using province specific weights since we combined data across provinces.

## Discussion

This study demonstrates the feasibility of using the best friend methodology to produce estimates of abortion incidence and the distribution of abortion methods and sources in two provinces in the DRC and reveals abortion is a common reproductive health event. Annual induced abortion incidence estimates were 17 per 1000 women of reproductive age in Kongo Central and 19 per 1000 in Kinshasa when examining respondent self-reported data, while rates were much higher – 44 and 105 abortions per 1000 women of reproductive age, respectively – when relying on adjusted friend data. Given concerns about under-reporting in self-reporting a sensitive behaviour like abortion, we view the friend estimates as more accurate, reflecting the high frequency of induced abortion in these settings. The more substantial gap in respondent versus friend estimates in Kinshasa compared to Kongo Central suggests greater stigma and reluctance to report one’s abortion to an interviewer in Kinshasa. This may be related to the fact that more women in Kinshasa were not acquainted with the interviewer than in Kongo Central and research suggests greater familiarity may lead to more disclosure of sensitive information in some instances.^37,38^ Additionally, we found women in Kongo Central were more likely to correctly report that the law allowed for legal abortion in certain circumstances, and generally had more accepting attitudes towards abortion than women in Kinshasa, which could result in a greater share of respondents who had an abortion reporting it on the survey in Kongo Central.

While no comparable abortion incidence estimates exist for Kongo Central, our Kinshasa abortion rate is nearly twice as high as prior estimates of 55 and 56 abortions per 1000 reproductive-aged women annually in 2015 and 2016, respectively.^6,7^ Some of this difference may be attributable to the increased use of induced abortion post-decriminalisation, but perhaps more important are the differences in methodologies. The AICM relied on knowledgeable informants to estimate the extent to which people who have abortions outside the formal healthcare system do not present for post-abortion care, but this may have been underestimated.^6^ Additionally, the study that used a social network-based method similar to ours, did not adjust for transmission bias (or any other biases).^7^ If the visibility of friends’ abortions in their study was similar to what we observed, their adjusted friend incidence rate for Kinshasa would have been 91 abortions per 1000 women aged 15–49 in 2015, pre-decriminalisation. This is much closer to our rate of 105 per 1000 in 2021 following decriminalisation and the increased availability of medical abortion pills in the private sector that has been observed over the past decade in the Global South.^39^

Our findings suggest women in Kinshasa and in Kongo Central may be approaching the management of their fertility in different ways. The abortion incidence was more than twice as high in Kinshasa compared to Kongo Central. Large differences between rural and urban settings are detected in other sub-Saharan Africa high fertility settings as well,^12,27,40^ highlighting the importance of abortion as a secondary strategy to manage one’s fertility in urban settings in particular. The abortion rate differences we observed in these two provinces are not explained by differences in the primary means of pregnancy prevention; in both Kinshasa and Kongo Central, a quarter of women use modern contraception while 11% and 18% have an unmet need for contraception, respectively.^41^ These contraceptive patterns result in high levels of unintended births in both settings (61% in Kinshasa and 62% in Kongo Central). However, fertility is much lower in Kinshasa than in Kongo Central (total fertility rates of 4.2 and 6.0, respectively),^42^ suggesting more women in Kinshasa than Kongo Central rely on abortion to achieve their desired fertility. This may be in part due to significant differences in the availability of abortion services; data from 2017 indicate 48% of facilities in Kinshasa had all components required to provide termination of pregnancy while only 35% of facilities in the region where Kongo Central is located met these criteria.^43^ Ensuring access to quality comprehensive abortion care is essential to achieving reproductive health and rights and enabling reproductive self-determination.

While we anticipated the friend abortion incidence estimates would be more accurate than respondent estimates, the comparative advantage of the friend data for examining abortion methods and sources was less obvious as respondents may be more likely to know about friend abortions that were less safe and resulted in complications; this pattern has been observed elsewhere.^27–29,31^ The distribution of abortions by whether they involved a recommended method and/or source was similar for respondents and friends in our data, but we view the respondent estimates as more accurate given the aforementioned concerns and the somewhat lower proportion of abortions involving non-recommended methods and sources in Kongo Central.

Inadequate safe abortion services and legal restrictions do not prevent abortions from happening but contribute to unsafe procedures. These unsafe procedures make up 88% of abortions in Central Africa, 78% of which are least safe.^2^ In the DRC, however, our results suggest that a much smaller proportion of women have potentially the least safe abortions as only 17% of respondents in Kinshasa and 30% in Kongo Central reported using non-recommended methods from non-recommended sources, which is roughly analogous to the WHO least safe criteria. These findings may reflect recent government reform to expand access to safe abortion. They may also result from the widespread use of medical abortion, which represents 30.7% of abortions in Kongo Central and 25.5% in Kinshasa, while specifically self-managed medical abortion accounts for 19.5% and 16.4% of abortions, respectively. The extent of medical abortions could be even higher if some of the 13.0% and 7.7% of women who used unknown pills actually used medical abortion pills; these abortions are currently categorised as non-recommended in our results. However, on the aggregate, we suspect our estimates of abortion involving recommended methods and sources *over*estimate the extent of abortion safety given evidence from many low-resource settings suggesting facility-based surgical abortions and self-managed medical abortions often do not adhere to medical guidelines.^44–50^ Additionally, there is selection bias in our abortion data since those who die of an unsafe abortion are inherently not in our sample, which would again overestimate abortion safety. This is especially true in a setting with high maternal mortality like the DRC where there were approximately 473 maternal deaths per 100,000 live births in 2017 and where cause-specific mortality estimates suggest 10% to 18% are due to unsafe abortion.^3,51,52^ The abortions involving non-recommended methods and sources, which likely drive the high abortion-related morbidity and mortality in the country, are more common in Kongo Central, suggesting the need to monitor policy implementation across DRC provinces, particularly in primary care settings in rural areas.

COVID-19 may have impacted abortion demand and care seeking in the DRC as the pandemic was ongoing through the period of interest. Research on general care seeking in the DRC suggests 54% of women who needed to visit a facility at the outset of COVID-19 had difficulty accessing care while evidence from North Kivu specifically indicated sexual and reproductive health service availability decreased initially but largely recovered by August 2020.^53,54^ Findings from other settings show decreases in abortion service availability and use at facilities during the pandemic, with evidence suggesting some resorted to self-managed abortion.^55^ COVID-19-related factors may have contributed to the level of self-managed medical abortion and the extent of non-recommended methods used in our study, which some healthcare workers and community members perceived to be the case, at least early in the pandemic.^56^ However, given there were no lockdowns in the DRC in 2021 and the DRC has had lower rates of infection and mortality than many parts of the world,^57^ we suspect women’s 2021 abortion trajectories were not substantially impacted by the pandemic.

These results need to be considered with the following limitations in mind. The best friend methodology has shown promise in improving abortion estimates by reducing social desirability bias. Nontheless, the method rests on three assumptions that need to be adjusted for if conditions are not met. First, the surrogate sample should be representative of women of reproductive age in the DRC. In our study, 37% of respondents reported having no close female friends, leading to potential selection bias in the surrogate sample. Comparisons between respondents and friends showed modest differences, which we attempted to account for by adjusting for “missing” friends and applying post-stratification weights. However, we cannot discount the potential for unobserved bias from unmeasured characteristics. Additionally, even after adjustments, a few small but significant differences remained between respondents and the friend sample.

Second, the best friend methodology assumes that respondents know about their best friend’s abortion experience. Assuming respondents share their own experience in the same way friends do, we found that 60% of respondents in Kinshasa and 55% in Kongo Central shared their abortion experience with their best friend. Correcting for this transmission bias increases friend abortion incidence rates from 64 abortions per 1000 to 105 in Kinshasa and from 25 to 44 in Kongo Central. Yet, the transmission bias adjustment may over- or under-adjust if the way we measure transmission does not accurately capture knowledge of this phenomenon between the friend dyads. Further work is needed to improve this adjustment. This could involve additional qualitative work to better understand the nature of communication on sensitive topics between friends. Additionally, investigators could use facility records to identify people who received facility-based abortion or post-abortion care to determine abortion sharing among those who report their abortion on a subsequent survey versus those who do not; interviewers could then ask about the abortion services received at the facility and whether their closest female friend knows about it among those who did not disclose it initially on the survey.

The last assumption is that reporting on one’s best friend reduces the social desirability bias. We found this to be true in the present study given the difference in estimates between self-reported versus unadjusted friend estimates of abortion incidence, though the confidence intervals did overlap for the estimates in Kongo Central given the uncertainty around these estimates. Our inclusion of menstrual regulation when worried about a pregnancy may lead to an overestimation of abortion if some women taking action to regulate their periods were in fact not pregnant. However, the diversification of language used to qualify an abortion was informed by prior quantitative and qualitative work suggesting women refer to abortion in different ways depending on the length of the pregnancy, the certainty of the pregnancy, and the methods used.^58,59^ The pilot we conducted prior to fieldwork in the DRC suggested this phenomenon was relevant in this context. Even if all pregnancies were not confirmed, women may experience complications if they rely on unsafe means to bring back their period.

Finally, the categorisation of abortions by whether they involved a recommended method and/or source is limited by the level of detail we could reliably collect from respondents on their and their friends’ abortions. It is likely that we have overestimated the extent of abortions involving recommended methods and sources given we do not have details on the specific abortion surgery or pill dosage/regimen and the conditions under which the abortion was performed. More broadly, evidence illustrates that even when an abortion does involve recommended methods and sources, it does not necessarily mean it was safe according to WHO guidelines; this applies to both facility-based surgical abortions as well as self-managed medical abortions.^44–50^ Additionally, it is important to look beyond safety to a rights-based conceptualisation of quality of care.^60^

In conclusion, this study shows that women in the DRC often rely on abortion to regulate their fertility and a significant proportion do so using non-recommended means, likely contributing to the country’s high levels of maternal morbidity and mortality. As such, significant work remains to actualise the commitments made in the Maputo Protocol to provide comprehensive reproductive health services that combine primary and secondary prevention services to reduce unsafe abortion and its negative sequelae.

## Data Availability

Data for this study are publicly available at pmadata.org; we relied on the Democratic Republic of the Congo Phase 3 female dataset. Anyone can access these data after completing a brief request form at https://www.pmadata.org/data/available-datasets.

## Footnotes

* We recognize that people other than those who identify as women can become pregnant and have abortions. We use the term “women” throughout this paper as that is the terminology the research we cite uses and our study survey included only people who identified as female.
